# Analysis of the Effects of Dietary Pattern on the Oral Microbiome of Elite Endurance Athletes

**DOI:** 10.3390/nu11030614

**Published:** 2019-03-13

**Authors:** Nida Murtaza, Louise M. Burke, Nicole Vlahovich, Bronwen Charlesson, Hayley M. O’Neill, Megan L. Ross, Katrina L. Campbell, Lutz Krause, Mark Morrison

**Affiliations:** 1 Faculty of Medicine, Translational Research Institute, University of Queensland Diamantina Institute, Brisbane, QLD 4102, Australia; nida.murtaza@uqconnect.edu.au (N.M.); l.krause@uq.edu.au (L.K.); 2 Centre for Exercise & Nutrition, Mary MacKillop Institute for Health Research, Australian Catholic University, Melbourne, VIC 3000, Australia; meg.ross@ausport.gov.au; 3 Australian Institute of Sport, Canberra, ACT 2617, Australia; Nicole.Vlahovich@ausport.gov.au (N.V.); bronwen.charlesson@outlook.com (B.C.); 4 Faculty of Health Sciences & Medicine, Bond University, Robina, QLD 4226, Australia; haoneill@bond.edu.au (H.M.O.); Katrina.Campbell@health.qld.gov.au (K.L.C.)

**Keywords:** oral microbiome, elite athletes, diet

## Abstract

Although the oral microbiota is known to play a crucial role in human health, there are few studies of diet x oral microbiota interactions, and none in elite athletes who may manipulate their intakes of macronutrients to achieve different metabolic adaptations in pursuit of optimal endurance performance. The aim of this study was to investigate the shifts in the oral microbiome of elite male endurance race walkers from Europe, Asia, the Americas and Australia, in response to one of three dietary patterns often used by athletes during a period of intensified training: a High Carbohydrate (HCHO; *n* = 9; with 60% energy intake from carbohydrates; ~8.5 g kg^−1^ day^−1^ carbohydrate, ~2.1 g kg^−1^ day^−1^ protein, 1.2 g kg^−1^ day^−1^ fat) diet, a Periodised Carbohydrate (PCHO; *n* = 10; same macronutrient composition as HCHO, but the intake of carbohydrates is different across the day and throughout the week to support training sessions with high or low carbohydrate availability) diet or a ketogenic Low Carbohydrate High Fat (LCHF; *n* = 10; 0.5 g kg^−1^ day^−1^ carbohydrate; 78% energy as fat; 2.1 g kg^−1^ day^−1^ protein) diet. Saliva samples were collected both before (Baseline; BL) and after the three-week period (Post treatment; PT) and the oral microbiota profiles for each athlete were produced by 16S rRNA gene amplicon sequencing. Principal coordinates analysis of the oral microbiota profiles based on the weighted UniFrac distance measure did not reveal any specific clustering with respect to diet or athlete ethnic origin, either at baseline (BL) or following the diet-training period. However, discriminant analyses of the oral microbiota profiles by Linear Discriminant Analysis (LDA) Effect Size (LEfSe) and sparse Partial Least Squares Discriminant Analysis (sPLS-DA) did reveal changes in the relative abundance of specific bacterial taxa, and, particularly, when comparing the microbiota profiles following consumption of the carbohydrate-based diets with the LCHF diet. These analyses showed that following consumption of the LCHF diet the relative abundances of *Haemophilus, Neisseria* and *Prevotella* spp. were decreased, and the relative abundance of *Streptococcus* spp. was increased. Such findings suggest that diet, and, in particular, the LCHF diet can induce changes in the oral microbiota of elite endurance walkers.

## 1. Introduction

Recent technological advances have enabled a more holistic definition and characterisation of the microbes that colonise the human body, the “microbiomes”. The human oral cavity serves as the habitat for a numerically large and diverse microbiome [1], which has been extensively characterised with respect to infectious and periodontal diseases, and caries, as well as for its contributions to the onset and progression of chronic conditions such as diabetes, cardiovascular disease and cancer [2]. However, the impacts of dietary pattern on the oral microbiome are not well defined, neither for the general population nor for cohorts who may follow specialised diets, such as elite athletes. In that context, recent studies have revealed a positive symbiotic association between the oral bacteria and host with respect to an enterosalivary nitrate-nitrite-nitric oxide pathway, which contributes to nitric oxide (NO) homeostasis [3,4]. Here, facultative anaerobic bacteria in the mouth reduce salivary gland concentrated nitrate to nitrite, which is then swallowed and absorbed into the bloodstream before further reduction to NO. The critical role of the oral microbiota in this effect has been demonstrated, where a seven-day period of antiseptic mouth wash treatment was shown to disrupt the oral microbiota of healthy non-athletes and, in the absence of any dietary modifications, was associated with reductions in plasma and oral nitrite levels and an increase in blood pressure [5]. These findings raise the spectre that diet may also invoke changes in the oral microbiota that manifest in alterations of this enterosalivary pathway and NO homeostasis but remains unexplored. 

A recent investigation [6] of the effect of diet and training on exercise metabolism and performance in elite endurance athletes provided an opportunity for pilot work on this theme. The “Supernova 1” study investigated parameters around endurance capacity in a cohort of elite endurance race walkers who followed one of the three popular dietary approaches during a three-week period of intensified training: a ketogenic Low Carbohydrate High Fat diet (LCHF), or a diet high in carbohydrates consumed either ad libitum (HCHO) or at specific periods on a daily/weekly basis (PCHO). While the HCHO diet is focused on optimal muscle and brain carbohydrate (CHO) stores for each training session, the PCHO diet involves a strategic combination of sessions with such dietary support as well as other which are undertaken with low muscle glycogen availability to promote greater metabolic stress and cellular adaptation [7,8]. Finally, the LCHF diet involves severe CHO restriction to promote adaptations that increase muscle capacity for fat oxidation [7,8]. Details on the rationale for these radically different types of nutrition [6,8] and the actual protocols employed in this study can be found elsewhere [6,7]. In summary, the Supernova 1 study found that each group of athletes achieved a significant improvement in their aerobic capacity over the training block, which was undertaken during the base phase of the annual training plan. However, while this was associated with improved economy and real-world race performance in the two groups who trained while consuming the HCHO and PCHO diets, the LCHF group experienced an increase in the oxygen cost of exercise supported by high rates of fat oxidation, and thereby failed to improve their race performance despite the gain in aerobic capacity [6]. Based on these differential results, the overall aim of this study was to examine whether and how the oral microbiome of these athletes was affected by their diet during intensified training. This appears to be the first study that provides an in-depth investigation of diet x oral microbiome interactions in elite athletes. 

## 2. Materials and Methods 

### 2.1. Study Design

The group of world-class race walkers and the design of the “Supernova 1” study are described in detail by Burke et al. [6] and Mirtschin et al. [7]. In summary, these male race walkers (aged 20–35 years, BMI range 16–23 kg/m^2^) were from Australia, Canada, Japan, Italy, Poland, Sweden, Chile and South Africa, and all met International Association of Athletics Federations (IAAF) standards for international competition, with more than 75% participating in the major championships during the year of the study (i.e., 2016 Rio Olympic Games and 2016 World Walking Cup). Twenty-nine study experiences were gained from 21 elite athletes who participated in either one (*n* = 13) or both (*n* = 8) of the Supernova 1 research camps conducted at the Australian Institute of Sport. Each camp involved three weeks of intensified training and rigorously supervised dietary interventions.

### 2.2. Allocation to Dietary Interventions 

The athletes were involved over several months of planning and received education about the range of likely effects of the diets on various aspects of health and performance. Each had ample time to choose a diet(s) according to his beliefs of the performance benefits from the chosen diet. Although this type of assignment was non-random, given that all athletes choose freely to be in the study and to be fed their diet of choice, this approach both promoted adherence to the intervention and controlled for the random effects of such rigorous dietary control (e.g., feeling anxious about losing personal freedom of dietary choice). Therefore, any effects on the oral microbiome could be attributed to the diet, including any additional intrinsic biochemical, physiological or psychological overlay that belongs to the diet itself. 

Three diets were compared: (i) a diet high in carbohydrate availability (HCHO; *n* = 9) comprised of 60% of energy intake from CHO (~8.5 g/kg body mass (BM)/day), 16% protein (~2.1 g/kg BM/day), 20% fat; (ii) a diet with periodised carbohydrate availability (PCHO; *n* = 10) of similar overall macronutrient composition as HCHO but consumed at different intervals across the day and throughout the week to support different training sessions with high or low CHO availability and (iii) a ketogenic low carbohydrate-high fat diet (LCHF; *n* = 10) comprised of 78% fat, 17% protein (~2.2 g/kg/day) and 0.50 g/kg/day carbohydrate (3.5% energy). All the meals were prepared taking into consideration the nutritional requirement within the allocated dietary intervention. Mirtschin et al. reported the detailed nutritional information and meal plans for the above three dietary interventions of this study [7]. 

### 2.3. Sample Collection, Genomic DNA Extraction and 16S rRNA Gene Amplicon Preparation

Saliva samples were collected from the athletes prior (baseline, BL) and after the three -week training-diet intervention using the OMNIgene saliva collection and preservative kit and according to the manufacturer’s instruction (fasted collection, saliva collected by spitting into the tube). Total DNA was extracted from 0.25 mL aliquots of the preserved saliva samples using the repeated bead-beating procedure for cell lysis [9] and an automated column-based DNA purification procedure (Maxwell^®^ 16MDx system, Promega Corporation, WI, USA) as described by Shanahan et al. [10]. Bar-coded PCR amplicon libraries of the V6-V8 hypervariable regions of 16S rRNA genes from Bacteria/Archaea were produced also following the protocols described by Shanahan et al. [10], and then sequenced via the Illumina MiSeq platform and workflows established by the Australian Centre for Ecogenomics at the University of Queensland.

### 2.4. Bioinformatics Analysis

The sequence data were analysed using the Quantitative Insights into Microbial Ecology (QIIME) software package on an Ubuntu Linux virtual machine. QIIME was used to demultiplex and perform quality control checking and filtering of the sequence data [11]. USEARCH 6.1 was used for the removal of candidate chimeric sequences [12]. The chimera checked filtered sequences were then clustered into Operational Taxonomic Units (OTUs) using the open reference OTU picking method. Threshold setting of 97% sequence identity was applied and Greengenes (version 13.8) database was used as the reference database [13]. Following the OTU picking step, OTUs that were not identified as Bacteria or Archaea, and/or OTUs that comprised ≤ 0.01% of the total sample sequence count, were discarded from further analysis. All of the samples with less than 1000 reads were also excluded from the OTU table. Single rarefaction was done by random sampling to the minimum read count (6861 reads) to generate a subsampled OTU table. 

### 2.5. Statistical Analysis

The rarefied OTU table was used to generate the taxonomy plots from phylum to genus levels and used to calculate alpha- and beta-diversity metrics. Alpha diversity was measured by Shannon index as an estimator for richness and evenness of microbiota communities. Weighted and unweighted UniFrac distance matrices were constructed in QIIME and used for Principle Coordinate Analysis (PCoA) of beta (between sample) diversity analysis. Redundancy Analysis (RDA) and Analysis of Similarity (ANOSIM) measures were also performed on these data within the Calypso web server to identify any clustering with respect to BL or the specific dietary patterns. Linear Discriminant Analysis (LDA) Effect Size (LEfSe) and sparse Partial Least Squares Discriminant Analysis (sPLS-DA) analyses via the Mixomics mixMC package within Calypso were then used to identify whether any individual taxa are discriminatory for the different dietary patterns [14,15]. The multiple linear regression analysis available via Calypso was used to perform pairwise comparisons between the BL and post-diet training microbiome profiles. A *p*-value < 0.05 was considered significant for all the statistical analysis. Tukey’s test was used to compare age and BMI data for the athletes assigned to each dietary group. 

### 2.6. Ethics Approval, Trial Registration and Consent to Participate

The study was approved by the Ethics committee of the Australian Institute of Sports (AIS, no. 20150802) and UQ-HREC 2015001965. The clinical trial is registered by the Australian New Zealand Clinical Trials Registry (ANZCTR) and has been assigned the number ACTRN12618001529235. The raw sequence paired-end files are deposited in the European Nucleotide Archive with the primary accession number PRJEB29801. All the subjects were informed and consented to do this research.

## 3. Results

As mentioned previously, 21 elite race walkers were recruited in the study (with eight athletes recruited in both the camps). Table 1 provides the anthropometric details of the subjects enrolled in the study. According to Tukey’s multiple comparisons test, there were no significant differences in the age (*p* = 0.53 for HCHO vs. PCHO; *p* = 0.28 for HCHO vs. LCHF and *p* = 0.87 for PCHO vs. LCHF) and the BMI scores (*p* = 0.36 for HCHO vs. PCHO; *p* = 0.84 for HCHO vs. LCHF and *p* = 0.67 for PCHO vs. LCHF) when athletes were grouped according to the dietary intervention they received. 

**Figure 1:**
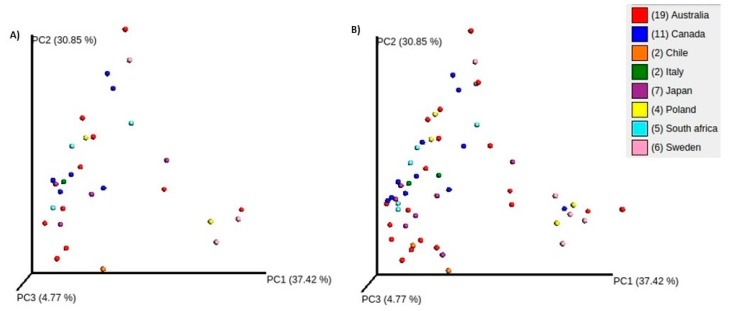
Principle component analysis of weighted UniFrac distances for the oral microbiomes of athletes at Baseline only (BL, **A**); and when combined with their profiles obtained after the diet-training intervention period (**B**). Samples are colored based on the athlete’s country of origin and show no significant clustering indicative of a dietary and/or ethnic effect on the oral microbiomes.

The Shannon alpha diversity was reduced following the diet-training interventions when compared to their subject-matched BL measures; however, these reductions were not statistically significant (*p* = 0.1 for BL vs. HCHO and BL vs PCHO; *p* = 0.62 for BL vs. LCHF). The PCoA analysis of the weighted UniFrac distances are shown in Figure 1 and did not reveal any distinct clustering of the saliva microbiome profiles, either with respect to the ethnic origin of the athletes or the dietary intervention. Similarly, the supervised analyses by RDA and ANOSIM did not identify any significant differences between the microbiota community composition at BL and following any of the three dietary interventions (data not shown). Taken together, these results suggest that the dietary interventions do not result in dramatic changes in the overall biodiversity of the oral microbiome, but rather more subtle changes in community composition. As mentioned previously in the Methods section, eight athletes were recruited in both the camps, and the two baseline profiles (B1 vs. B2) of these eight athletes were compared. No substantive differences between the two microbiota profiles were apparent, as assessed by Shannon alpha-diversity and ANOSIM beta-diversity analyses. These tests indicate that the time between the study camps was sufficiently long to ensure a “washout” between the two camps, and, thereby, no potential carryover effects from the previous diet on the subsequent results/profiles.

### 3.1. Comparisons of Community Profiles of Saliva Samples between Baseline and Post Interventions

**Figure 2:**
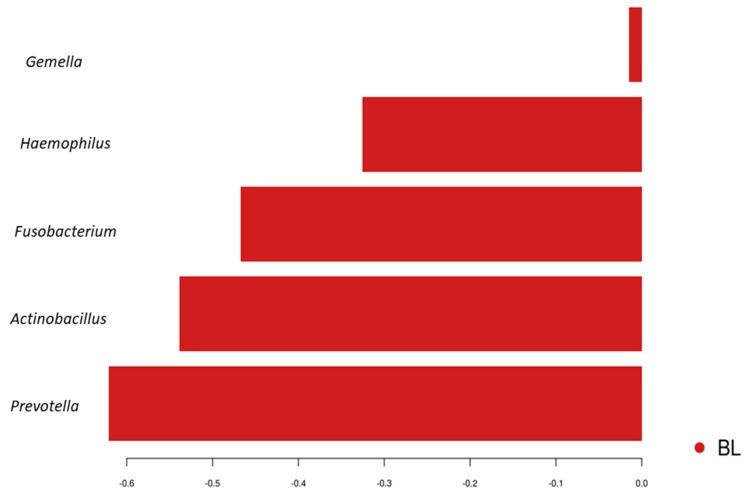
Genera differentiating between the oral microbiota profiles of athletes at baseline (BL, red) and after their consumption of the High Carbohydrate diet (HCHO) identified by sparse Partial Least Squares Discriminant Analysis (sPLS–DA).

LefSe analyses was used to identify discriminating taxa between baseline (BL) and post diet-training interventions. OTU’s affiliated to *Streptococcus*, *Peptostreptococcus*, *Actinomyces*, *Granulicatella*, *Atopobium*, *Veillonella* and *Prevotella* were found to be enriched following the consumption of HCHO diet, whereas *Parvimonas* was discriminatory and enriched for the BL samples from these same athletes (Supplementary Figure S1). Analysis by the sPLS-DA of the same athlete samples identified *Prevotella, Actinobacillus*, *Fusobacterium*, *Haemophilus* and *Gemella* to be associated and increased in BL samples (Figure 2). Pairwise comparisons (Supplementary Figure S2) of the oral microbiota profiles at BL and following HCHO diet training intervention was also examined using mixed effect linear regression, and the relative abundance of *Atopobium* was found to increase (*p* = 0.015), whereas *Capnocytophaga* (*p* = 0.027) and *Porphyromonas* (*p* = 0.03) were decreased after consumption of HCHO diet, when compared to the BL. However, no significant differences were observed once correction for multiple testing using false discovery rate was applied (FDR = 0.49).

**Figure 3:**
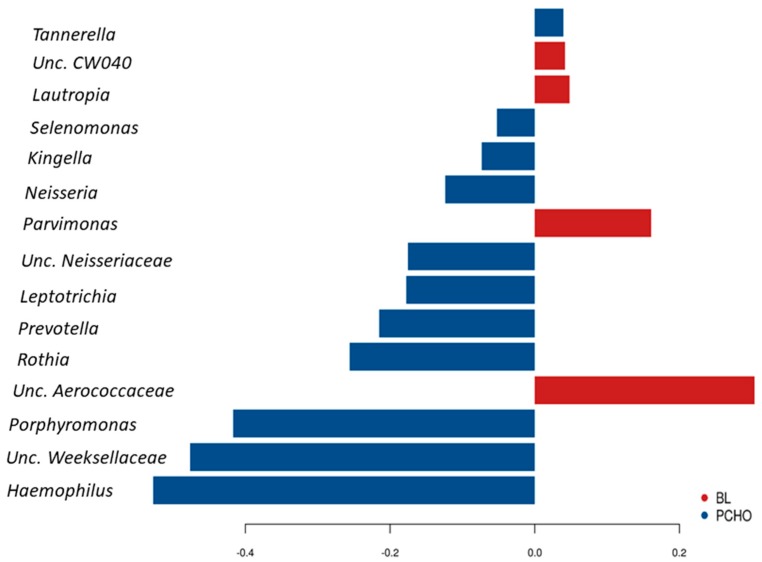
Genera differentiating between the oral microbiota profiles of athletes at baseline (BL, red) and after their consumption of the Periodised Carbohydrate diet (PCHO, blue) identified by sparse Partial Least Squares Discriminant Analysis (sPLS–DA).

LefSe analysis was then used to compare the microbiota profiles between BL and PCHO diet and revealed that the OTU’s affiliated with *Leptotrichia, Neisseria, Moryella and Actinomyces* to be discriminatory and enriched for BL, whereas OTU’s affiliated with *Streptococcus*, *Kingella*, unclassified members of *Neisseriaceae* and *Prevotella* were increased and discriminatory in the same athletes after consumption of the PCHO diet (Supplementary Figure S3). Analysis by sPLS-DA further showed that the relative abundances of *Haemophilus, Neisseria, Porphyromonas, Leptotrichia, Kingella, Prevotella, Unclassified Neisseriaceae, Rothia, Selenomonas and Tannerella* were increased following the consumption of the PCHO diet *whereas Unc. Aerococcaceae, Unc. CW040, Lautropia and Parvimonas* were distinct and increased in the BL samples of the same athletes who later received the PCHO dietary intervention (Figure 3). Repeated measures mixed effect linear regression analysis showed that the genus *Actinomyces* (*p* = 0.04), *Moryella* (*p* = 0.05), *Oribacterium* (*p* = 0.04), *Peptostreptococcus* (*p* = 0.009) and some unclassified *Erysipelotrichaceae* (*p* = 0.04) were reduced in response to the PCHO diet as compared to BL (Supplementary Figure S4). However, the statistical significance of all these differences was lost once correction for multiple testing using the false discovery rate was applied (FDR = 0.4) (Supplementary Figure S4). 

**Figure 4:**
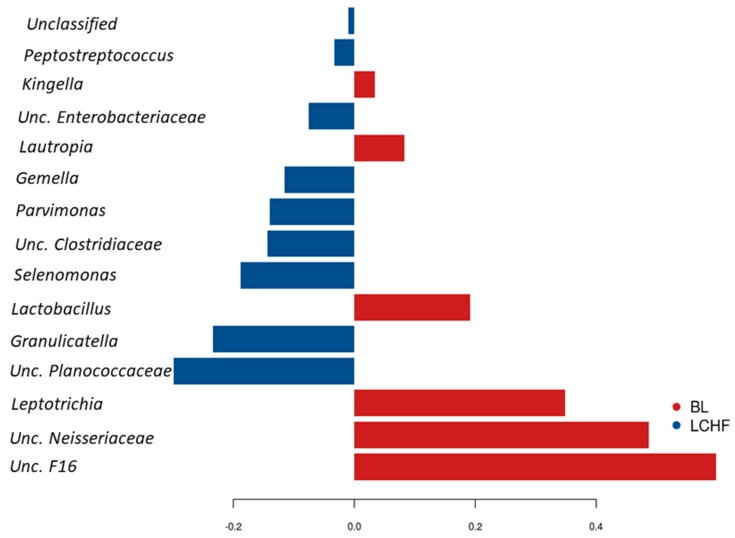
Genera differentiating between the oral microbiota profiles of athletes at baseline (BL, red) and after their consumption of the Low Carbohydrate High Fat diet (LCHF, blue) identified by sparse Partial Least Squares Discriminant Analysis (sPLS–DA).

Discriminating taxa for BL and for the same samples following the LCHF diet. Training intervention was also identified using LefSE and sPLS-DA. LefSe analysis revealed *Leptotrichia*, *Lachnospiraceae* and *TM-7* affiliated OTU’s to be increased in the BL samples, whereas *Lactobacillales*, *Streptococcus*, *Neisseria* affiliated OTU’s were discriminatory and increased in the LCHF group (Supplementary Figure S5). The sPLS-DA identified *Selenomonas, Unc. Planococcaceae, Unc. Enterobacteriaceae, Peptostreptococcus, Gemella, Granulicatella, Parvimonas Unc. Clostridiaceae* to increase following LCHF diet training intervention, whereas *Unc. F16*, *Unc. Neisseriaceae, Leptotrichia*, *Lactobacillus*, *Lautropia* and *Kingella* to be distinct and enriched in the BL samples of same athletes (Figure 4). According to repeated measures analysis using mixed effect linear regression, the genus *Fusobacterium* (*p* = 0.02), *Lautropia* (*p* = 0.05), *Aggregatibacter* (*p* = 0.04), *Leptotrichia* (*p* = 0.040) and some unclassified *F16* (*p* = 0.04) were reduced, whereas *Granulicatella* (*p* = 0.03), some unclassified Planococcaceae (*p* = 0.03) and *Streptococcus* (*p* = 0.048) were increased in response to LCHF diet when compared to microbiota profiles at their BL (Supplementary Figure S6). However, no significant differences were observed once correction for multiple testing using false discovery rate was applied (FDR = 0.4).

### 3.2. Comparisons of Community Profiles of Saliva Samples at the Conclusion of Dietary Interventions 

**Figure 5:**
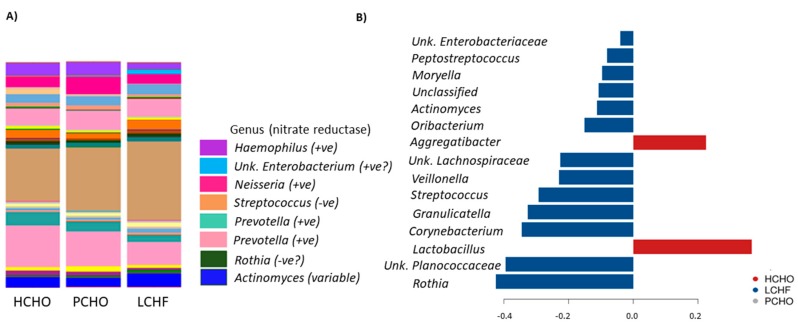
Oral microbiome profiles (genus-level) of athletes consuming either a high carbohydrate (HCHO), periodised carbohydrate (PCHO) or a low-carbohydrate high-fat diet (LCHF) after the diet-training intervention where bar plots represent: (**A**) relative abundance of genera in saliva samples after dietary interventions and their inferred nitrate reductase activity; (**B**) microbial families associated with different diets as identified by sparse Partial Least Squares Discriminant Analysis (sPLS-DA).

Figure 5 summarises the results of these analyses, showing the community profiles present in saliva samples at the conclusion of the dietary intervention periods, with annotations around some key genera and their inferred nitrate reductase capacity (Figure 5A). These profiles were compared with each other using sPLS-DA, which can be used to extract those taxa that most strongly discriminate the community structure between treatment groups (Figure 5B). Longitudinal comparison of the taxonomic profiles in the samples using sPLS-DA (Figure 5B) showed the strongest effect of the LCHF dietary intervention and in particular increase in the abundance of Gram-positive (Firmicutes) bacteria such as *Streptococcus*, *Peptostreptococcus*, and *Rothia*. 

**Figure 6:**
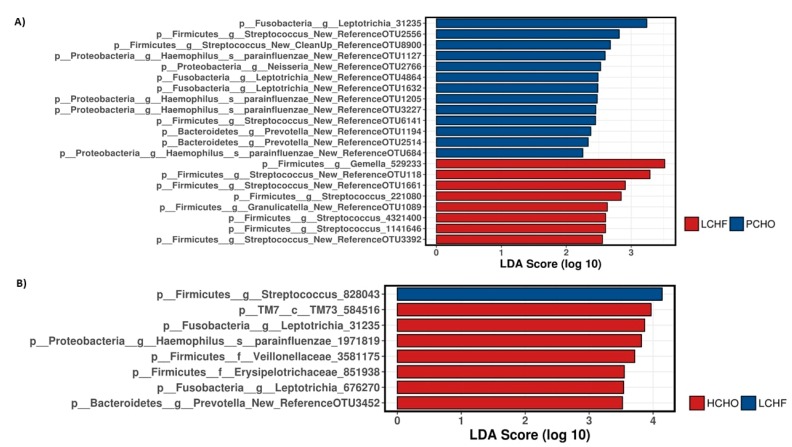
Linear Discriminant Analysis (LDA) Effect Size (LefSe) analysis at Operational Taxonomic Unit (OTU) level to compare the oral microbiome profiles of athletes post training diet interventions between Periodised Carbohydrate/Low Carbohydrate High Fat diet (PCHO/LCHF) (**A**) and High carbohydrate-Low Carbohydrate high-fat diet (HCHO/LCHF) (**B**), respectively.

LefSe analyses was also used to examine the differences in oral microbiomes post intervention and these analyses showed that the discriminating and enriched taxa (at the OTU level) were *Streptococcus* affiliated OTUs for the LCHF diet intervention, whereas Gram-negative bacteria (e.g., *Haemophilus* and *Leptotrichia* spp.) were among the enriched and discriminating taxa for the HCHO/PCHO diets (Figure 6A,B). 

## 4. Discussion

Despite the relatively small number of participants in this study, the dietary pattern consumed by these elite athletes during intensified training was shown to invoke remarkable effects on specific oral bacterial taxa—the bacterial communities in the mouth. The lack of a matching cohort of non-athletes (non-race walkers) and the lack of comprehensive data on the habitual dietary intake of athletes (i.e., BL samples) are acknowledged but were beyond the logistical and financial scope of the trial design. Furthermore, but understandably, the elite nature of the athletes ensured the group size is quite small, which also reduces the power needed for stringent statistical tests of significance, or the further subgrouping of the athletes according to ethnicity, etc. However, and despite these limitations, this is the first study of its type with elite endurance athletes, and one of the very few studies of the diet/nutrients × oral microbiota interactions affecting the physiology and/or metabolism of healthy human subjects. Furthermore, while fluctuations in the abundance and/or activities of nitrate-reducing bacteria in the oral microbiome are recognised to affect an individual’s responsiveness to nitrate supplementation [16], this is the first study of the effects of dietary manipulation on this specific microbiome. 

The bacterial taxa found in this study are similar to those represented in the Human Oral Microbiome Database (HOMD), as well as those typically reported in other studies of mainstream human subjects [17,18]. The beta-diversity UniFrac principal coordinates analysis showed no apparent clustering of the oral microbial communities based on the ethnicity of the athletes nor any distinct effects of the dietary interventions under investigation in this study. This is similar to the findings of other studies of healthy individuals in which no significant clustering and bacterial taxa changes in the oral cavity have been reported [17]. Nevertheless, more subtle changes within the bacterial communities in association with the diets were observed, with some of these representing potential alterations in community–host symbiosis. Here, the comparisons of the oral microbiome collected after three weeks of consuming one of three widely used diets by elite athletes during intensified training revealed that, unlike the CHO-rich diets, a ketogenic-LCHF diet appears to shift the balance of bacterial taxa that are widely considered to be key governors of the enterosalivary nitrate-nitrite-nitric oxide (NO) axis within the oral cavity. This is an important finding since previous studies have demonstrated functional effects on host health when alterations to the oral microbiome interfere with this pathway [19]. Facultative anaerobic bacteria in the mouth reduce salivary gland concentrated nitrate to nitrite, which is then swallowed and absorbed into the bloodstream, before further reduction to NO [3,4,20]. 

The critical role of the oral microbiome in this effect has been recently demonstrated in healthy non-athletes, where a seven-day period of antiseptic mouth wash treatment was shown to disrupt the oral microbiota and, in the absence of any dietary modifications, was associated with reductions in plasma and oral nitrite levels and an increase in blood pressure [20]. Taken together, the changes seen following consumption of the LCHF diet, with respect to reductions in the relative abundances of well-known Gram-negative nitrate/nitrite reducers such as *Haemophilus*, *Prevotella* and *Neisseria*; and an increase in *Streptococcus* spp., which are not recognised to be directly involved in nitrate/nitrite reduction; raises the spectre that consumption of the LCHF diet can impair the enterosalivary nitrate-nitrite-NO axis. Indeed, further indirect support for this hypothesis can be found in a recent brief report that a three-day LCHF diet was associated with an impaired plasma nitrate/nitrite conversion following supplementation with potassium nitrate, compared with the response observed when people consumed a HCHO diet [21]. This suggests that a LCHF diet might alter the baseline contribution of the nitrate-nitrite-NO pathway to NO-related health and performance benefits in athletes, as well as reduce their responsiveness to nitrate/beetroot juice supplementation as a performance aid [22]. Further supporting evidence comes from the major outcome of the Supernova 1 study, which is the primary study to the current project and from which these saliva samples were derived [6]. The study found a reduction in exercise economy (i.e., an increased oxygen cost of exercise) across a range of walking speeds in the LCHF group. It was originally hypothesised that this contributed to the failure of the LCHF group to improve their performance of a 10,000 m race walking event, despite the improvement in aerobic capacity that was seen across each of the study groups in response to the three week block of intensified training It is plausible to attribute this loss of economy to the substantial increase in the contribution of fat oxidation to exercise substrate needs in the LCHF group, noting the longstanding recognition that CHO oxidation is slightly more economical in generating ATP than fat oxidation per unit of oxygen utilization [23]. Additionally, the increase in exercise tolerance and performance following acute and/or chronic nitrate supplementation include improved oxygen delivery to the muscle via the vasodilatory effects of NO, as well as a direct effect on mitochondria to reduce proton leak [22]. However, these benefits are not universally observed across and within studies, and this variability is partially attributed to individual responsiveness, in addition to the more obvious contribution of unsuitable study protocols in relation to both the supplementation and exercise elements. However, based on the findings reported here, it is also plausible that part of the reduced exercise efficiency observed in the Supernova 1 study might be attributed to an altered oral microbiome, resulting in a reduction in nitrate/nitrite reducing activity and NO generation, with coordinate effects on circulation and mitochondrial function.

## 5. Conclusions

In conclusion, the results presented here are the first direct comparison of the oral microbiota profiles of elite athletes, and the effects of the dietary pattern consumed during intensified training for race-walking. The LCHF diet resulted in the most dramatic effects on the oral microbiota, with reductions in the relative abundance (*Haemophilus*, *Neisseria* and *Prevotella)*, and with a coincident increase in the relative abundance of *Streptococcus* spp. The athletes participating in this study following consumption of the LCHF diet also showed a loss of exercise economy (i.e., an increased oxygen cost of exercise) across a range of walking speeds compared to athletes consuming the carbohydrate rich diets [6]. The findings reported here therefore justify the need to examine how diet x oral microbiome interactions affect elite athlete performance; and, particularly, NO homeostasis, and any coordinate impacts on cardiovascular and circulatory physiology. 

## Figures and Tables

**Table 1:** Athlete cohort characteristics.

	High Carbohydrate (HCHO) Diet	Periodised Carbohydrate (PCHO) Diet	Low Carbohydrate High Fat (LCHF) Diet
Sample size	*n* = 9	*n* = 10	*n* = 10
Age (years)	25.4 ± 4	27.4 ± 4.6	28.3 ± 3.5
BMI (kg/m^2^)	20 ± 1.6	21 ± 1.3	20.4 ± 1.8
Country of origin	Australia, Canada, Japan, South Africa	Australia, Canada, Japan, Poland, Sweden, Italy	Australia, Canada, Japan, Poland, Sweden, Chile, South Africa
Gender	Male	Male	Male

Note: Data for Age and body mass index (BMI) are shown as mean ± standard deviation.

## Supplementary Materials

The following are available online https://www.mdpi.com/2072-6643/11/3/614/s1, Figure S1: Genera differentiating between the oral microbiota profiles of athletes at baseline (BL, red) and after their consumption of the High Carbohydrate diet (HCHO, blue) identified by LefSE; Figure S2: Mixed effect linear regression identified significant reductions in the relative abundances of *Capnocytophaga* (*p* = 0.027) and *Porphyromonas* (*p* = 0.032) whereas significant increase in the relative abundance of *Atopobium* (*p* = 0.015) after consumption of the High carbohydrate (HCHO) diet. Relative abundance was compared by mixed effect linear regression, including sampling time point as fixed effect and athlete as random effect. BL: baseline. Samples collected from the same individual are connected by lines; Figure S3: Genera differentiating between the oral microbiota profiles of athletes at baseline (BL, red) and after their consumption of the Periodised Carbohydrate diet (PCHO, blue) identified by LefSE; Figure S4: Mixed effect linear regression identified significant reductions in the relative abundances of *Actinomyces* (*p* = 0.043), *Moryella* (*p* = 0.053), *Oribacterium* (*p* = 0.042), *Peptostreptococcus* (*p* = 0.009) and *Unc. Erysipelotrichaceae* (*p* = 0.042) after consumption of the Periodised carbohydrate (PCHO) diet. Relative abundance was compared by mixed effect linear regression, including sampling time point as fixed effect and athlete as random effect. BL: baseline. Samples collected from the same individual are connected by lines; Figure S5: Genera differentiating between the oral microbiota profiles of athletes at baseline (BL, red) and after their consumption of the Low Carbohydrate High Fat diet (LCHF, blue) identified by LefSE; Figure S6: Mixed effect linear regression identified significant reductions in the relative abundances of *Fusobacterium* (*p* = 0.020), *Lautropia* (*p* = 0.048), *Leptotrichia* (*p* = 0.040), *Aggregatibacter* (*p* = 0.044) and *Unc. F16* (*p* = 0.040) whereas significant increase in the relative abundances of *Granulicatella* (*p* = 0.038), *Streptococcus* (*p* = 0.048) and *Planococcaceae* (*p* = 0.038) after consumption of the low carbohydrate high fat (LCHF) diet. Relative abundance was compared by mixed effect linear regression, including sampling time point as fixed effect and athlete as random effect. BL: baseline. Samples collected from the same individual are connected by lines.
