# Supplementary material for: Analysis of the Effects of Dietary Pattern on the Oral Microbiome of Elite Endurance Athletes
Source: Nutrients. 2019 Mar 13;11(3):614. doi: 10.3390/nu11030614 (PMC6471070; doi:10.3390/nu11030614)
Supplement: Supplementary file 1 [file nutrients-11-00614-s001.zip › Supplementaryfiles/Supplementaryfigures_legends.docx]

**Supplementary figures**

**Supplementary figure 1**


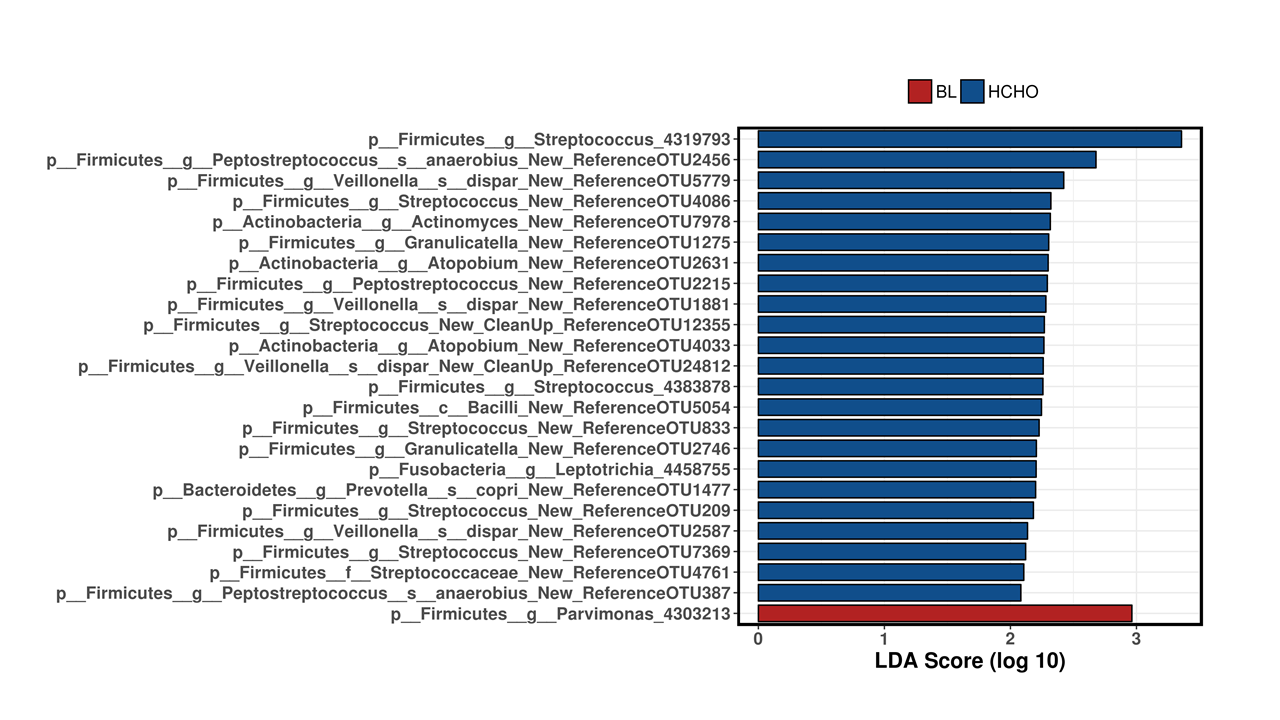


**Figure S1:** Genera differentiating between the oral microbiota profiles of athletes at baseline (BL, red) and after their consumption of the High Carbohydrate diet (HCHO, blue) identified by LefSE.

**Supplementary figure 2**

**
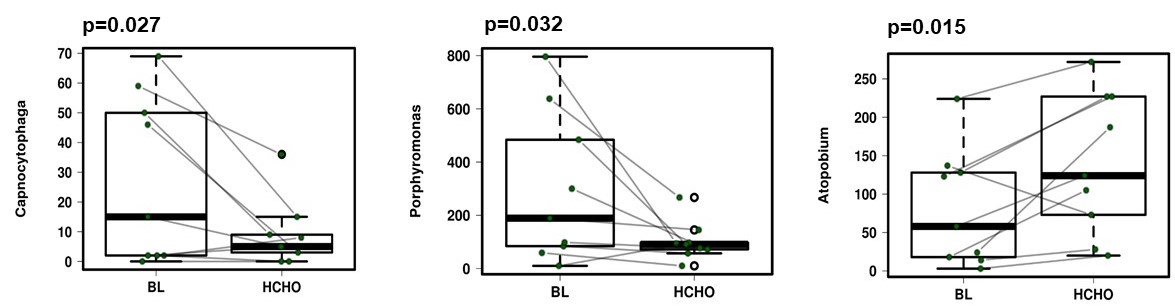
**

**Figure S2**: Mixed effect linear regression identified significant reductions in the relative abundances of *Capnocytophaga* (p=0.027) and *Porphyromonas* (p=0.032) whereas significant increase in the relative abundance of *Atopobium* (p=0.015) after consumption of the High carbohydrate (HCHO) diet*.* Relative abundance was compared by mixed effect linear regression, including sampling time point as fixed effect and athlete as random effect. BL: baseline. Samples collected from the same individual are connected by lines.

**Supplementary figure 3**


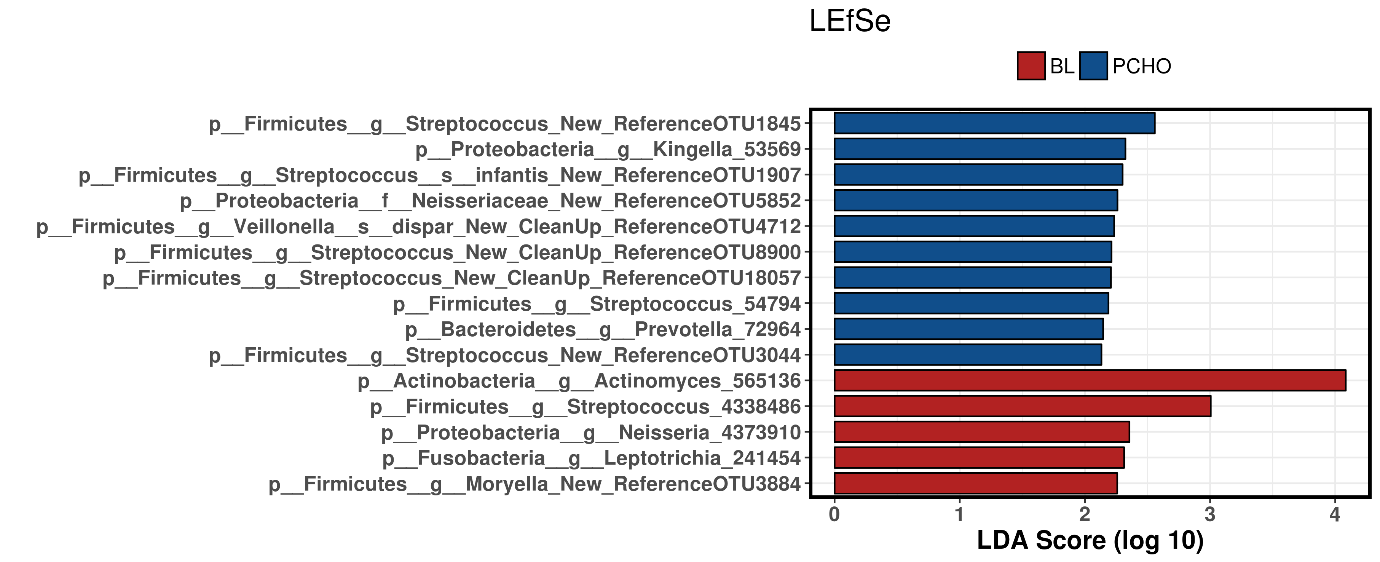


**Figure S3:** Genera differentiating between the oral microbiota profiles of athletes at baseline (BL, red) and after their consumption of the Periodised Carbohydrate diet (PCHO, blue) identified by LefSE.

**Supplementary figure 4**


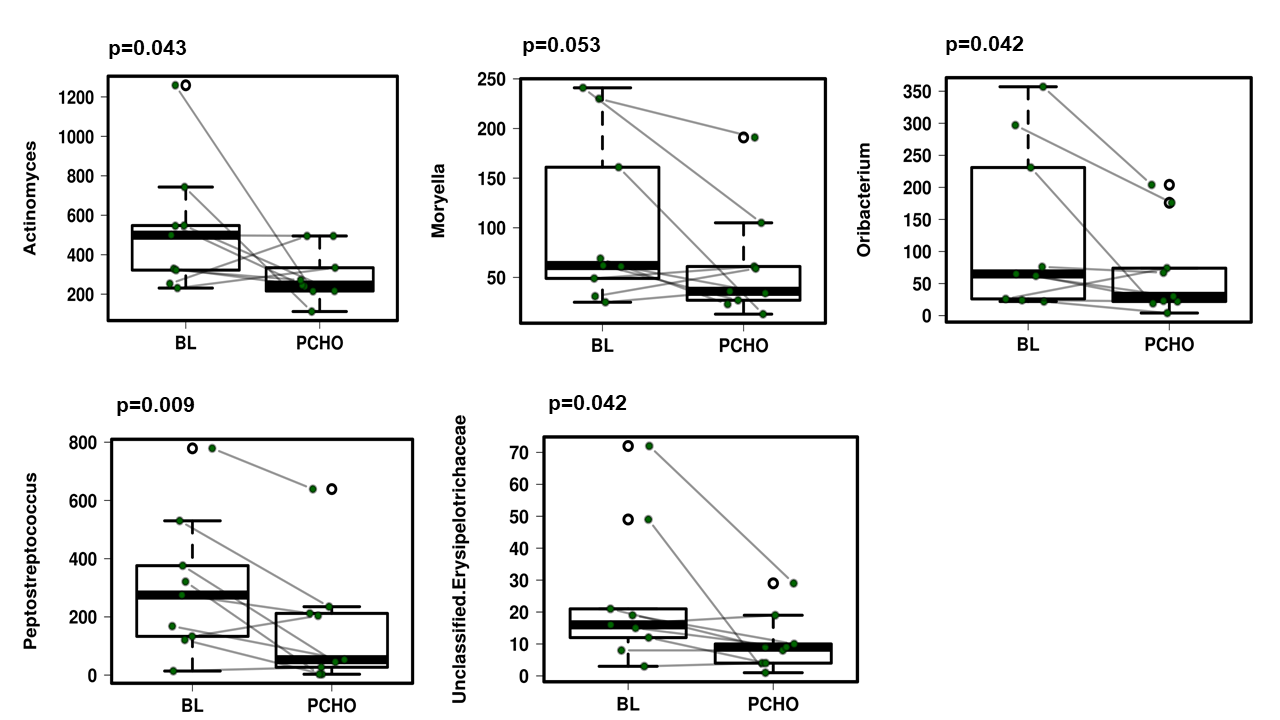


**Figure S4**: Mixed effect linear regression identified significant reductions in the relative abundances of *Actinomyces* (p=0.043), *Moryella* (p=0.053), *Oribacterium* (p=0.042), *Peptostreptococcus* (p=0.009) and *Unc. Erysipelotrichaceae* (p=0.042) after consumption of the Periodised carbohydrate (PCHO) diet*.* Relative abundance was compared by mixed effect linear regression, including sampling time point as fixed effect and athlete as random effect. BL: baseline. Samples collected from the same individual are connected by lines.

**Supplementary figure 5**


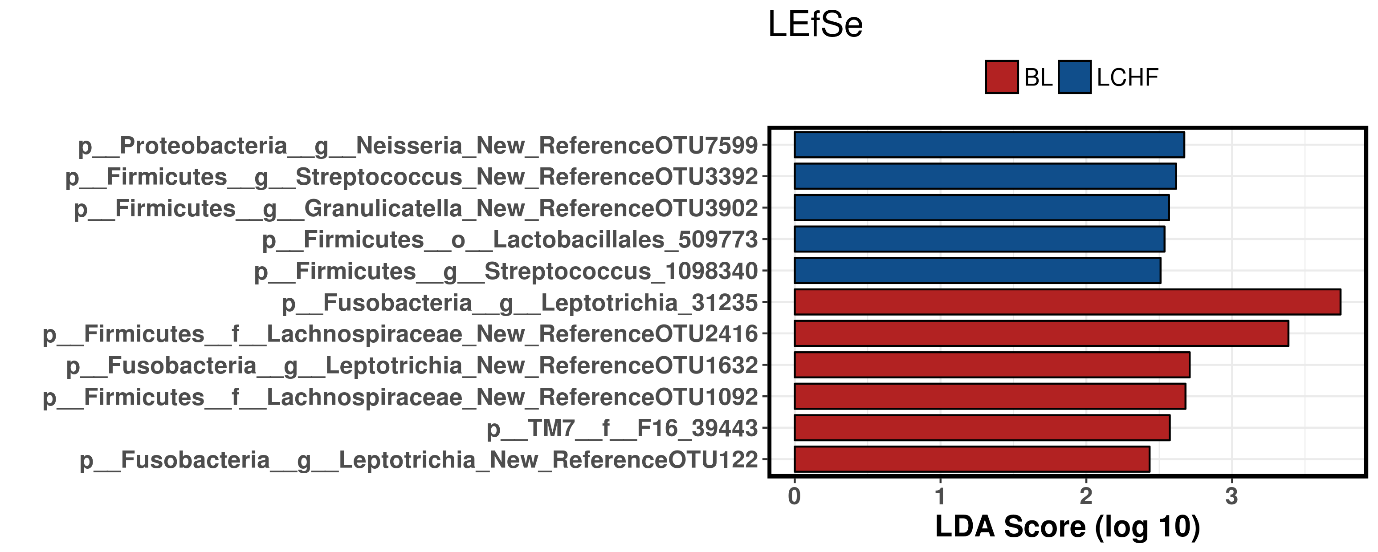


**Figure S5:** Genera differentiating between the oral microbiota profiles of athletes at baseline (BL, red) and after their consumption of the Low Carbohydrate High Fat diet (LCHF, blue) identified by LefSE.

**Supplementary figure 6**


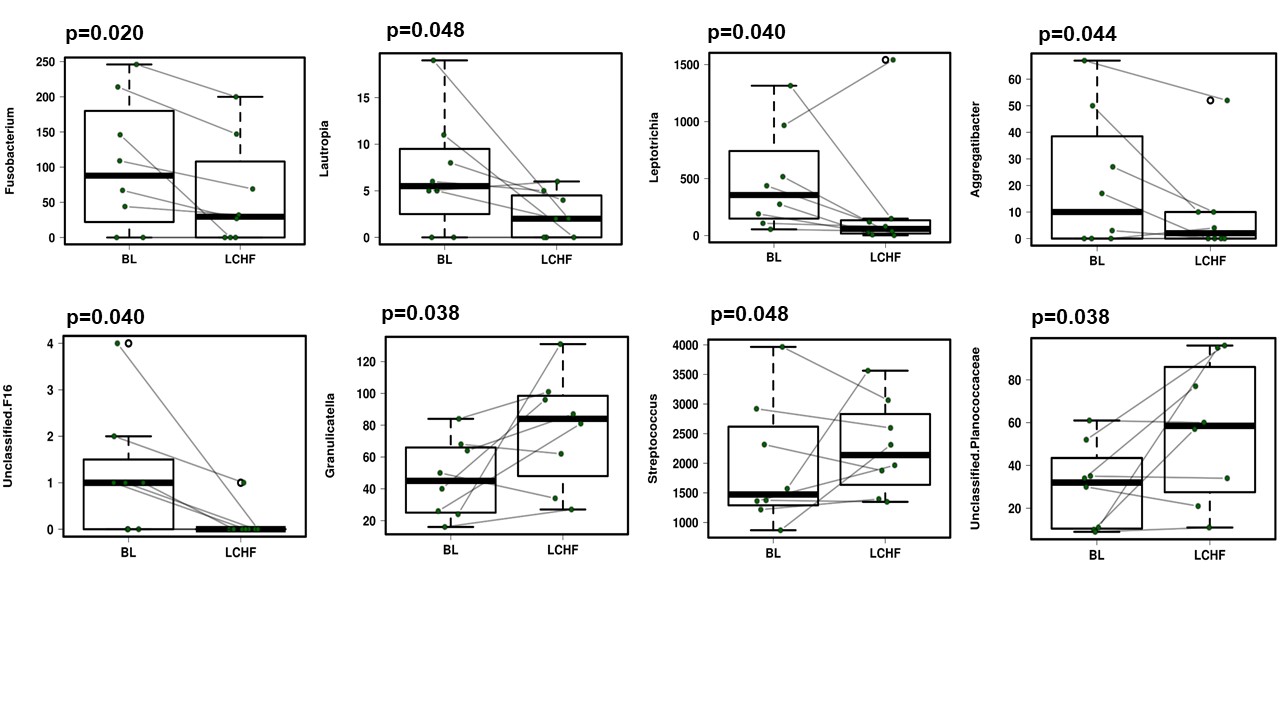


**Figure S6**: Mixed effect linear regression identified significant reductions in the relative abundances of *Fusobacterium* (p=0.020), *Lautropia* (p=0.048), *Leptotrichia* (p=0.040), *Aggregatibacter* (p=0.044) and *Unc. F16* (p=0.040) whereas significant increase in the relative abundances of *Granulicatella* (p=0.038), *Streptococcus* (p=0.048) and *Planococcaceae* (p=0.038) after consumption of the low carbohydrate high fat (LCHF) diet*.* Relative abundance was compared by mixed effect linear regression, including sampling time point as fixed effect and athlete as random effect. BL: baseline. Samples collected from the same individual are connected by lines.
