# Supplementary figures and images for: Analysis of the Effects of Dietary Pattern on the Oral Microbiome of Elite Endurance Athletes
Source: Nutrients. 2019 Mar 13;11(3):614. doi: 10.3390/nu11030614 (PMC6471070; doi:10.3390/nu11030614)

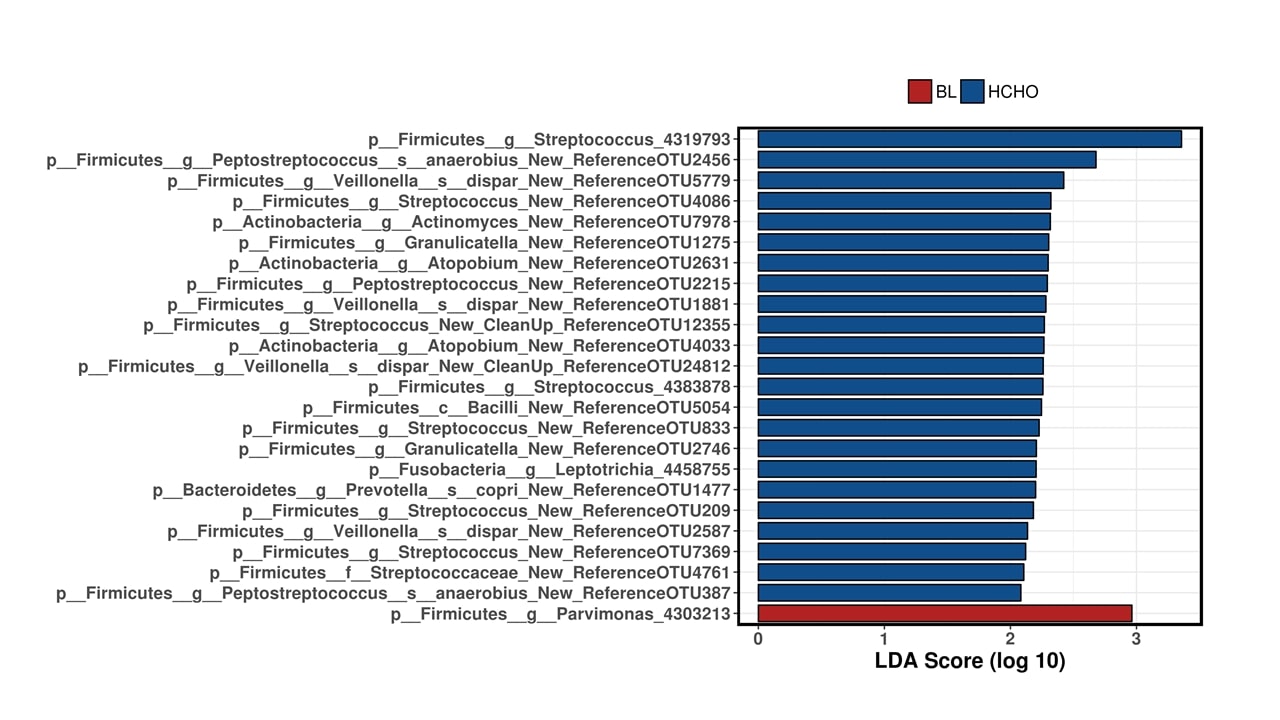

Supplement: Supplementary file 1 [file nutrients-11-00614-s001.zip › Supplementaryfiles/Suppfigure1.jpg]

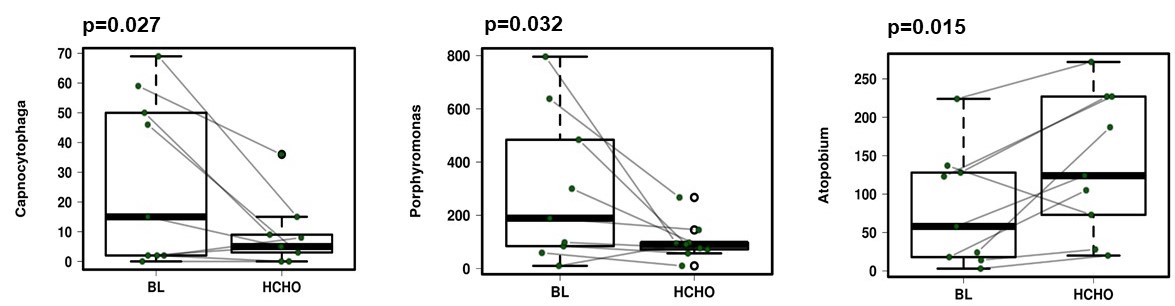

Supplement: Supplementary file 1 [file nutrients-11-00614-s001.zip › Supplementaryfiles/Suppfigure2.jpg]

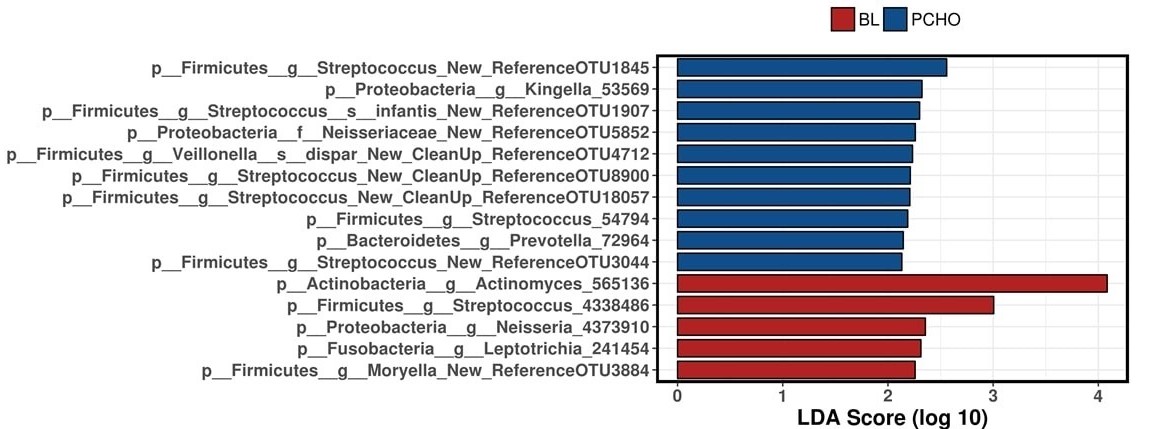

Supplement: Supplementary file 1 [file nutrients-11-00614-s001.zip › Supplementaryfiles/Suppfigure3.jpg]

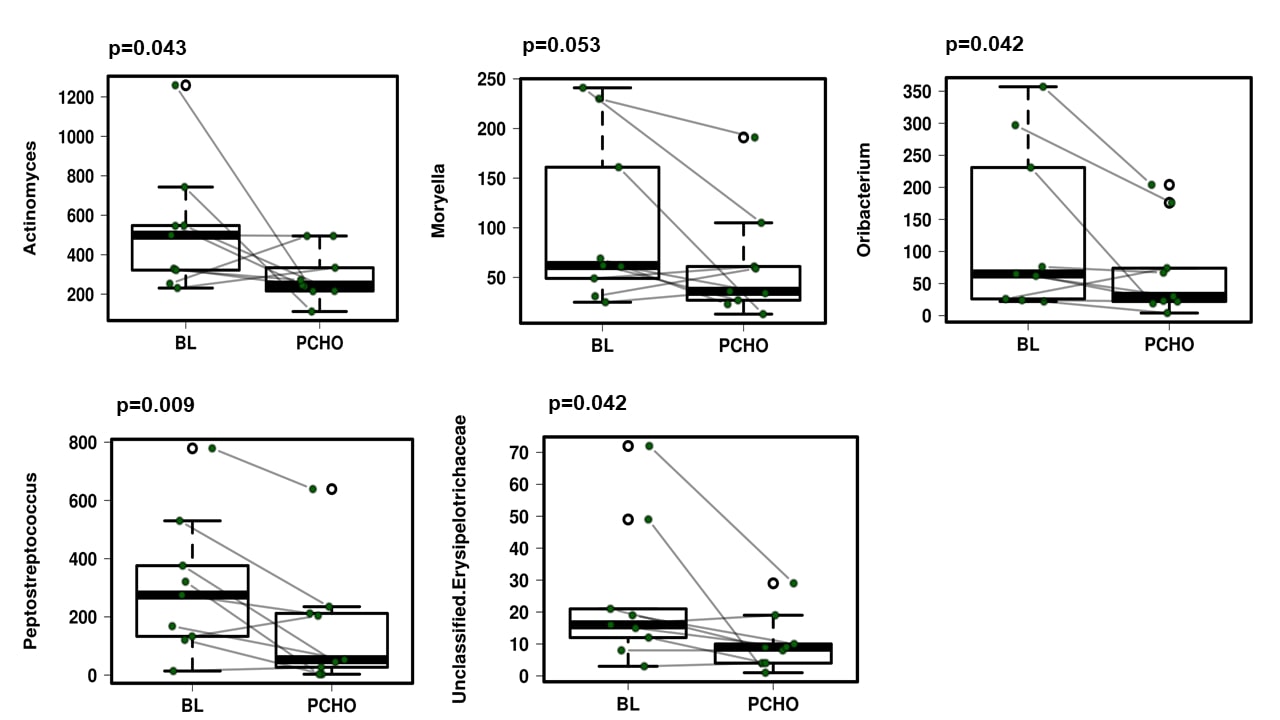

Supplement: Supplementary file 1 [file nutrients-11-00614-s001.zip › Supplementaryfiles/Suppfigure4.jpg]

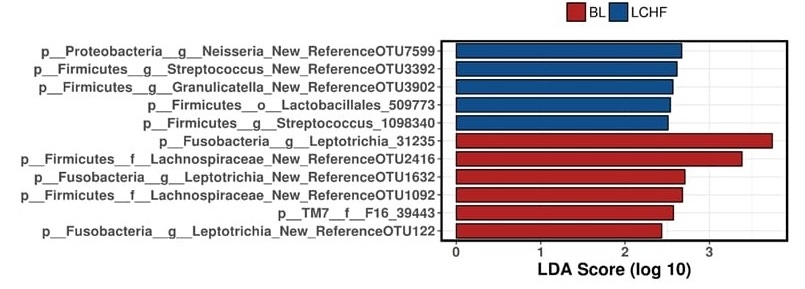

Supplement: Supplementary file 1 [file nutrients-11-00614-s001.zip › Supplementaryfiles/Suppfigure5.jpg]

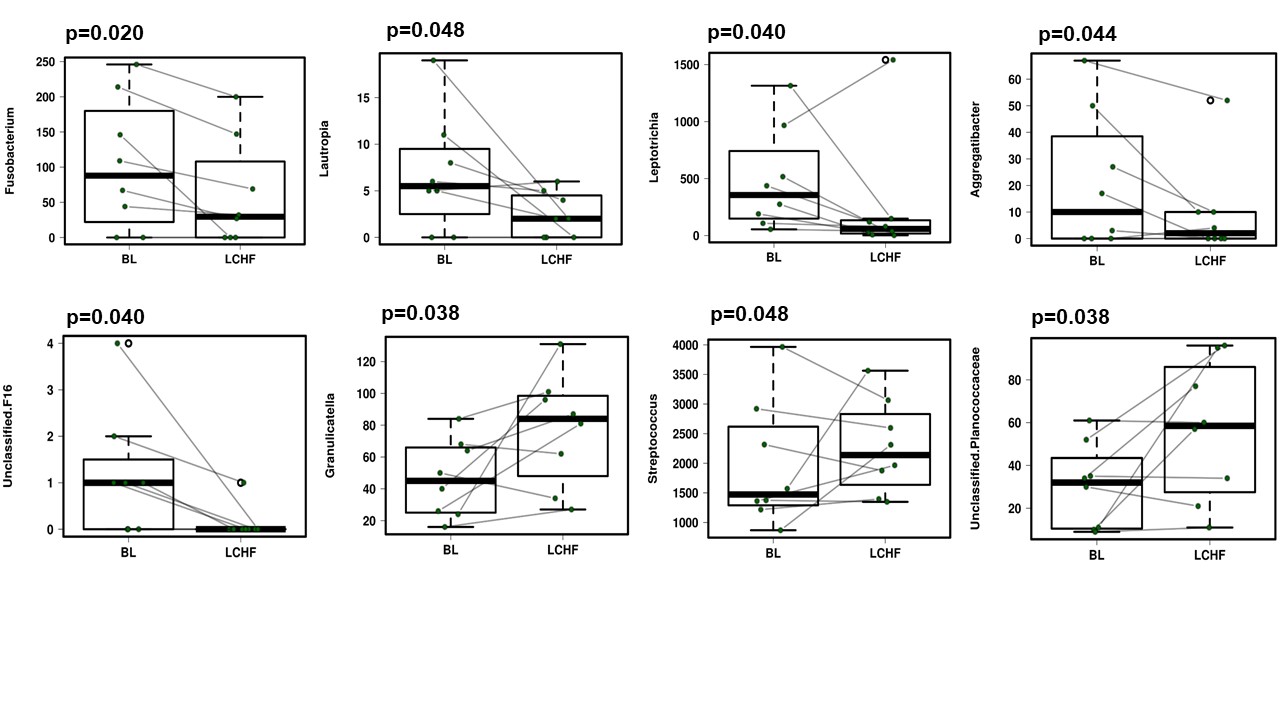

Supplement: Supplementary file 1 [file nutrients-11-00614-s001.zip › Supplementaryfiles/Suppfigure6.jpg]
